# Outcomes following external beam radiotherapy to the prostate and pelvic lymph nodes in addition to androgen deprivation therapy in non-metastatic prostate adenocarcinoma with regional lymph node involvement: a retrospective cohort study

**DOI:** 10.1259/bjro.20220030

**Published:** 2023-03-01

**Authors:** Akmal Mohamad Roji, Rahul Sandhu, Anjali Zarkar

**Affiliations:** 1 University Hospitals Birmingham NHS Foundation Trust, Birmingham, United Kingdom

## Abstract

**Objective:**

There is a paucity of evidence for external beam radiotherapy (EBRT) in patients with non-metastatic prostate adenocarcinoma with regional lymph nodes (cN1) as primary treatment in addition to androgen deprivation therapy (ADT). We present the retrospective outcomes of cN1 patients treated with prostate and pelvic nodal (PPLN) EBRT and ADT.

**Methods:**

The clinical records of cN1 patients given PPLN EBRT from January 2012 to January 2020 were retrospectively reviewed. Primary outcomes of overall survival, prostate cancer-specific survival, and failure-free survival were analysed. Secondary outcomes of biochemical relapse-free survival, locoregional recurrence-free survival, and distant metastases-free survival were also reviewed. The prognostic values of clinicopathological parameters were investigated. Treatment toxicity was also reviewed.

**Results:**

We identified 121 cN1 patients treated with PPLN EBRT and ADT. Treatment was well tolerated, with only a minority (1.7%) having Grade 3 toxicities. 5-year overall survival and prostate cancer-specific survival were 74.4 and 89.1% respectively. 5-year failure-free survival was 55.4%; with 5-year biochemical relapse-free survival, locoregional recurrence-free survival, and distant metastases-free survival at 56.2%, 85.2%, and 65.4% respectively. The benefits of PPLN EBRT were seen in most patients, with prolonged failure-free period and good loco-regional control.

**Conclusion:**

Patients with cN1 disease should be considered for PPLN EBRT, in addition to ADT. Treatment is well tolerated with low toxicity, good locoregional control, and prolonged time to disease progression.

**Advances in knowledge:**

We report real-world experience of cN1 patients treated with PPLN EBRT in addition to ADT, with good outcomes following treatment and low toxicity.

## Introduction

At diagnosis, 5–10% of high-risk prostate adenocarcinomas have synchronous nodal metastases on CT.^1^ The incidence is probably much higher, as nodal metastases detection increases to 30% using prostate-specific membrane antigen positron-emission tomography (PSMA-PET).^2,3^ The management of non-metastatic prostate adenocarcinoma with regional nodal metastases (cN1 patients) is controversial. Some consider pelvic lymph node (PLN) involvement as a marker of systemic disease requiring systemic therapy whilst others deem this as locoregional spread suitable for local treatment such as definitive external beam radiotherapy (EBRT). There is a lack of prospective evidence to guide management in this cohort. The Radiation Therapy Oncology Group (RTOG) 96–08 study, the only randomised study evaluating EBRT in combination with androgen deprivation therapy (ADT) in cN1 patients, closed prematurely due to poor accrual. Recently, STAMPEDE published a retrospective analysis of their cN1 patient cohort, showing prolonged failure-free survival (FFS) with EBRT.^4^ STAMPEDE has already recommended EBRT for cN1 patients,^5^ with consistent dose, fractionation, and treatment volumes reported. Beyond this, there have been no recent published prospective evidence of EBRT in this patient group.

As a result, much of the evidence in this area is from non-randomised retrospective analyses. However, there exists marked heterogeneity in patient selection within these publications. Many do not describe the EBRT volumes and techniques, with only a few describing EBRT of the PLN in addition to the prostate and seminal vesicles (SVs). Here, we add to the evidence base by describing the outcomes of our cN1 patients treated with prostate and pelvic nodal (PPLN) EBRT within a large hospital within the United Kingdom (UK) using a standard dose fractionation regime in addition to ADT.

## Methods

### Patients

The study was approved by the local audit management board, and patient details were anonymised during data collection and analysis. Data were collected on consecutive cN1 patients referred for PPLN EBRT between January 2012 and January 2020. Patients were referred from within the regional cancer network, covering a population of approximately 2.2 million people.^6^ Patients referred for EBRT following prostatectomy, those who had received upfront systemic therapy, and those involved in clinical trials were excluded from analyses.

### Staging and pathology

Disease staging followed the seventh edition of the AJCC/UNM staging.^7^ All patients had work-up of localised high risk prostate adenocarcinoma; including baseline prostate-specific antigen (PSA), MRI of the pelvis, staging CT, and bone scan. Patients of cN0 or M1 status were excluded from analyses. PLN were considered involved if >10 mm in diameter in the short axis on conventional radiological imaging and following consensus review by our urology multidisciplinary team.

### Hormonal therapy

All cN1 patients were started on neo-adjuvant ADT for up to 6 months with a luteinizing hormone-releasing hormone (LHRH) agonist. An anti-androgen was also prescribed for the initial 4 weeks from LHRH agonist initiation. Patients were then consented for delayed PPLN EBRT up to 6 months to allow for maximal cytoreduction. After EBRT, ADT was continued until to 2–3 years.

### External beam radiotherapy planning and delivery

For EBRT planning, the prostate, SV, and PLN were delineated on the planning system as described by consensus guidance.^8,9^ Briefly, for the PLN EBRT volumes, the pelvic iliac vessels were delineated from the lower border of the L5 vertebra down to the obturators stopping 10 mm above the pubic symphysis. This is then expanded circumferentially by 7 mm, editing off muscle and bone, to create the PLN clinical target volume (CTV), and further expanded globally by 5 mm to create the PLN planning target volume (PTV). For the organs at risk (OARs), the anal canal, rectum, bladder, large bowel, small bowel, and both right and left femoral heads were outlined. Patients were asked to keep an empty rectum and comfortably full bladder during CT simulation and EBRT.

EBRT was delivered using intensity modulated radiotherapy (IMRT) via Tomotherapy. As per the PIVOTAL trial^8^ (NCT00444821—a Phase II feasibility study of EBRT to the prostate, SV, and PLN *vs* EBRT to the prostate and SV in high risk cN0 patients) and STAMPEDE trial^10^ protocols, the PLN were treated up to 55–60 Gy in 37 fractions (38–55 Gy in EQD2), whilst the prostate and SV were treated up to 74 Gy in 37 fractions (74 Gy in EQD2). All EBRT volumes were treated together via simultaneous integrated boost (SIB) aiming to deliver 95% dose coverage of the PTV. All patients received EBRT as per pre-defined protocols to ensure dose constraints for the OARs were met.^8^

### Follow-up

Patients were reviewed during EBRT to assess and manage toxicities. Acute and chronic toxicities were recorded as described by the RTOG and European Organisation for Research and Treatment of Cancer.^11^ Acute toxicity was defined as adverse effects recorded during EBRT and up to 3 months afterwards, whilst chronic toxicities were defined as adverse effects recorded beyond 3 months.

On completion of EBRT, as per NICE 2008 guidelines,^12^ and later the West Midlands Expert Advisory Group guidelines,^13^ clinical follow-up consisted of review at 3-, 6-, and 12 months post-treatment. Subsequent reviews were scheduled every 6 months for the second and third year, and annually afterwards. Reviews consisted of clinical assessment, PSA testing, and toxicity assessment for erectile dysfunction and urinary and/or bowel incontinence.

### Outcomes

The primary outcomes included 5-year failure-free survival (FFS), overall survival (OS), and prostate cancer-specific survival (PCSS). FFS was defined as the time from PPLN EBRT to the first of either biochemical recurrence, locoregional recurrence, distant metastases, or death from prostate cancer. OS was defined as time from PPLN EBRT until death from any cause, whilst PCSS was defined as time from PPLN EBRT until death from prostate cancer, as recorded within clinical records or death certificates. Patients still alive were censored at last follow-up.

Secondary outcomes included 5-year biochemical relapse-free survival (BRFS), locoregional recurrence-free survival (LRFS), and distant metastases-free survival (DMFS). BRFS was defined as time from PPLN EBRT until biochemical relapse according to the Radiation Therapy Oncology Group–Association of Therapeutic Radiation Oncology (RTOG-ASTRO) Phoenix Consensus Conference Definition (nadir PSA+2).^14^ LRFS was defined as the time from PPLN EBRT until detection of recurrent disease within the EBRT field, either at the primary site or locoregional PLN, on radiological imaging. DMFS was defined as the time from PPLN EBRT until detection of recurrent disease beyond the pelvis in other organ sites on radiological imaging. Patients who were failure-free were censored at last follow-up.

### Statistical analysis

Data were presented as frequencies, means, and median with ranges. Results of primary and secondary outcomes were displayed using Kaplan–Meier curves, and survival estimates at specific time points were derived from life tables. Comparisons using univariate cox proportional hazard model analyses were conducted to investigate prognostic factors. Threshold for categorical variables were determined following review of the literature to allow comparison (Table 1). This included patient characteristics (including age and WHO performance status), tumour characteristics (including T stage, number of nodes, Gleason grade grouping, and initial & nadir PSA values), and treatment received (including PLN EBRT dose and length of adjuvant ADT). The values derived from statistical analysis were two-sided and a *p*-value of <0.05 was considered statistically significant. Data were analysed using the open source JAMOVI (v. 2.0) statistical software.

**Table 1.** Studies investigating outcomes of cN1 patients treated with PPLN EBRT

Study	Patient numbers	ADT	Pelvic node EBRT dose (EQD2)	Locoregional relapse	Distant metastases	FFS	OS	Comments
**Retrospective data**								
Lilleby et al (2015) Norway	Total = 136 †cN1 = 71	All had 2.5 years	43.5 Gy	*All patients: 19/136 (13.9%)	*All patients: 19/136 (13.9%)	5-year FFS *All patients: 76.2%	5-year OS *All patients: 89% †cN1 patients: 96.5%	*Results included high risk cN0 patients and not separated out. †Only cN1 patients <75 years with <3 lymph nodes involved were included in the study.
Tsumura et al (2018) Japan	Total = 40 cN1 = 22 M1 = 18	All had at least 36 months	50 Gy	*Not stated:	*Not stated	5-year FFS *All patients: 64.4%	5-year PCSS *All patients: 87.9%	*16/40 (40%) developed disease progression. Results included oligometastatic M1 patients and not separated out. All patients had high-dose rate brachytherapy to prostate followed by local EBRT boost.
Mallick et al (2019) India	Total = 61	All had 2 to 3 years	45.8 Gy *(SIB up to 62–72 Gy)	4/61 (6.6%)	11/61 (18%)	4-year FFS 77.5%	4-year OS 91%	*Patients treated with IMRT and had pathological nodes treated up to 60 Gy in 20 fractions.
Ieiri et al (2020) Japan	Total = 93	Not stated	41–50 Gy†	*Not stated	*Not stated	Median FFS 99 months	Median OS 130 months	*36/93 (38.7%) developed disease progression. Median FU of living patients at censorship = 56 months. †Fractionation regime of EBRT not described.
Tsuchida et al (2020) Japan	Total = 51	66.7% over 12 months	45% >60 Gy 55% <60 Gy	5/51 (10.0%)	15/51 (29.0%)	7-year FFS ≥60 Gy 90.6% <60 Gy 58.0%	7-year PCSS ≥60 Gy 95.0% <60 Gy 87.7%	Study looked at dose escalation to involved pelvic lymph nodes.
Our study (2022) Birmingham	Total = 121	76.9% had ≥24 months	38–55 Gy (treated via SIB)	15/121 (12.4%)	39/121 (32.2%)	5-year FFS 55.4%	5-year OS 74.4% 5-year PCSS 89.1%	cN1 patients included in analysis with up to five or more lymph nodes involved.
**Prospective data**								
RTOG 85–31 cN1 subset analysis (2005)	*Total = 173 EBRT + ADT = 98	Duration not stated	44–46 Gy (in 1.8 to 2 Gy per fraction)	EBRT + ADT = 25/98 (26%)	EBRT + ADT = 32/98 (33%)	5-year PSA control < 1.5 ng ml^−1^ EBRT + ADT = 54%	5-year OS EBRT + ADT = 72%	*Included patients treated with prostatectomy (*n* = 42). Lymph nodes were pathologically staged.
STAMPEDE N1M0 subset analysis (2016)	Total = 177 ADT + EBRT = 71	At least 2 years or until progression	3D-CRT 45–50 Gy IMRT 50 Gy	20/177 (11.3%) *Not stated for cN1 patients having EBRT	22/177 (12.4%) *Not stated for cN1 patients having EBRT	Estimated 5-year FFS ADT + EBRT = 65%	Estimated 5-year OS ADT + EBRT = 82%	Subset analysis using cohort study data from STAMPEDE control arm. *Only 58 of the 71 patients reporting EBRT received treatment to both prostate and pelvis nodes.

ADT, androgen deprivation therapy; EBRT, external beam radiotherapy; FFS, failure-free survival; FU, follow-up; IMRT, intensity modulated radiotherapy; OS, overall survival; PCSS, prostate cancer-specific survival; PPLN, prostate and pelvic nodal; SIB, simultaneous integrated boost.

## Results

### Patient characteristics

**Figure 1.**
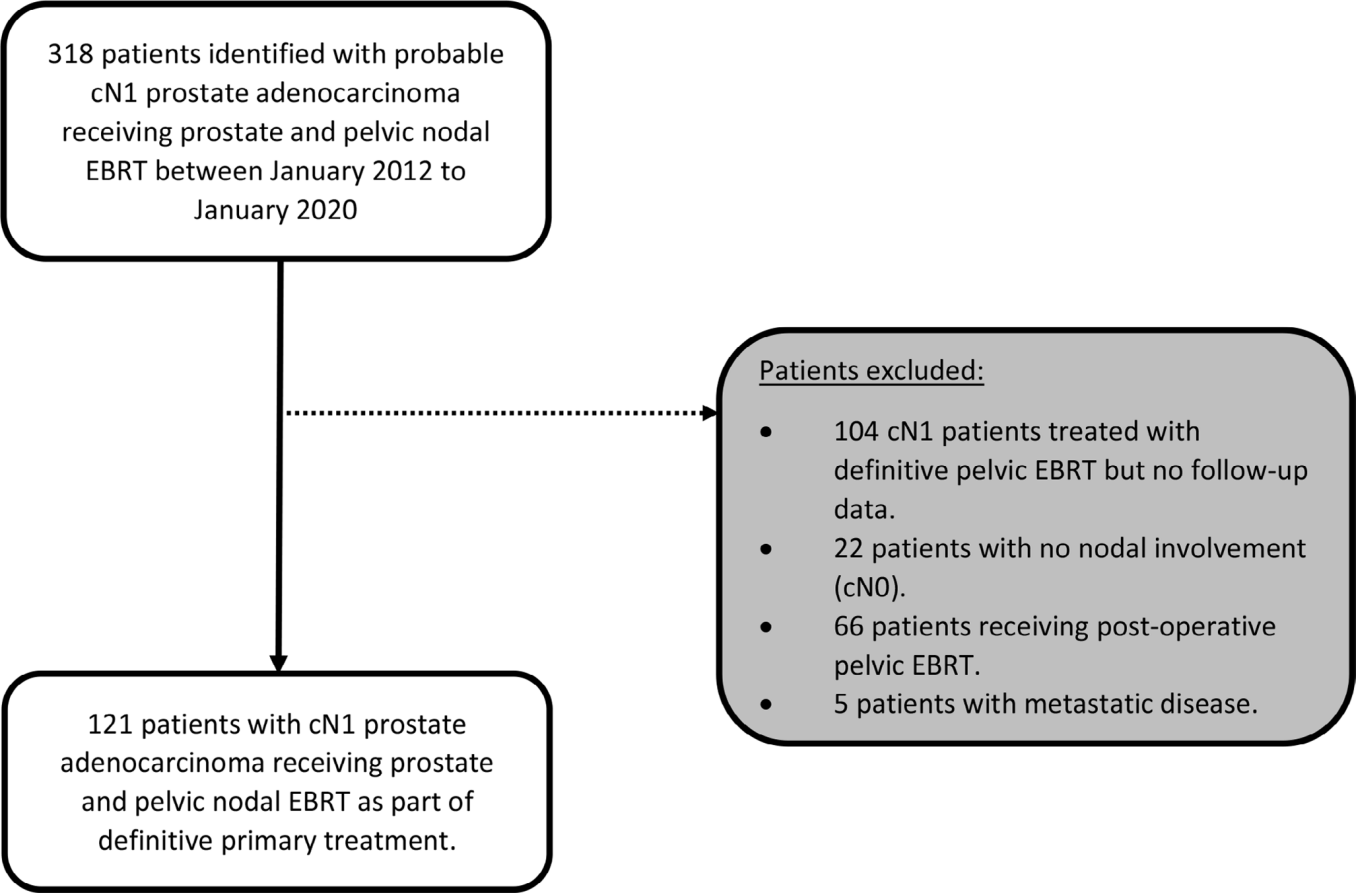
Patient selection flow diagram. EBRT, external beam radiotherapy.

During the period studied, a total of 318 patients were identified to have received PPLN EBRT from January 2012 to January 2020. Following review, 197 patients were excluded (Figure 1) due to the following: cN1 patients treated with PPLN EBRT but no follow-up data (*n* = 104), cN0 patients (*n* = 22), post-operative EBRT (*n* = 66), and metastatic disease (*n* = 5). The remaining 121 cN1 patients were included in the final analysis. Demographics, tumour characteristics, and treatment details are summarised in Table 2. Mean PSA before treatment was 32 ng ml^−1^ (range 3–514), with most presenting with T3 disease (62.8%) and Gleason grade grouping 4 to 5 (71.1%). Majority of patients (76.9%) received a minimum of 2 years of ADT post-EBRT.

**Table 2.** Patient characteristics

Characteristics	
**Number of patients**	121
**Patient follow-up (months)**	
Median	56
Range	(1 – 145)
**Age (years)**	
Median	69
Range	52–83
**Gleason grade grouping (%)**	
1–3	34 (28.1%)
4–5	86 (71.1%)
Unknown	1 (0.8%)
**World Health Organisation performance status**	
0	94 (77.7%)
≥ 1	26 (21.5%)
Unknown	1 (0.8%)
**PSA before ADT (ng/mL)**	
Median (range)	32 (3 – 514)
**Primary tumour (T)**	
T1 to T2	34 (28.1%)
T3	76 (62.8%)
T4	11 (9.1%)
**Size and number of pelvic nodes involved** Median size (range)	12 mm (4–30 mm)
Up to two lymph nodes involved	84 (69.4%)
From three to four lymph nodes involved	19 (15.7%)
From five or more lymph nodes involved	12 (9.9%)
Unknown	6 (5.0%)
**Length of ADT**	
Less than 1 year	8 (6.6%)
From 1 to 2 years	20 (16.5%)
From 2 to 3 years	91 (75.2%)
More than 3 years	2 (1.7%)
**Total EBRT dose to pelvic nodes (in EQD2, with SIB to prostate & seminal vesicles)**	
<43 Gy	2 (1.7%)
43–54 Gy	58 (47.9%)
≥55 Gy	59 (48.8%)
Unknown	2 (1.7%)

ADT, androgen deprivation therapy; EBRT, external beam radiotherapy; PSA, prostate-specific antigen.

### Radiotherapy treatment

**Figure 2.**
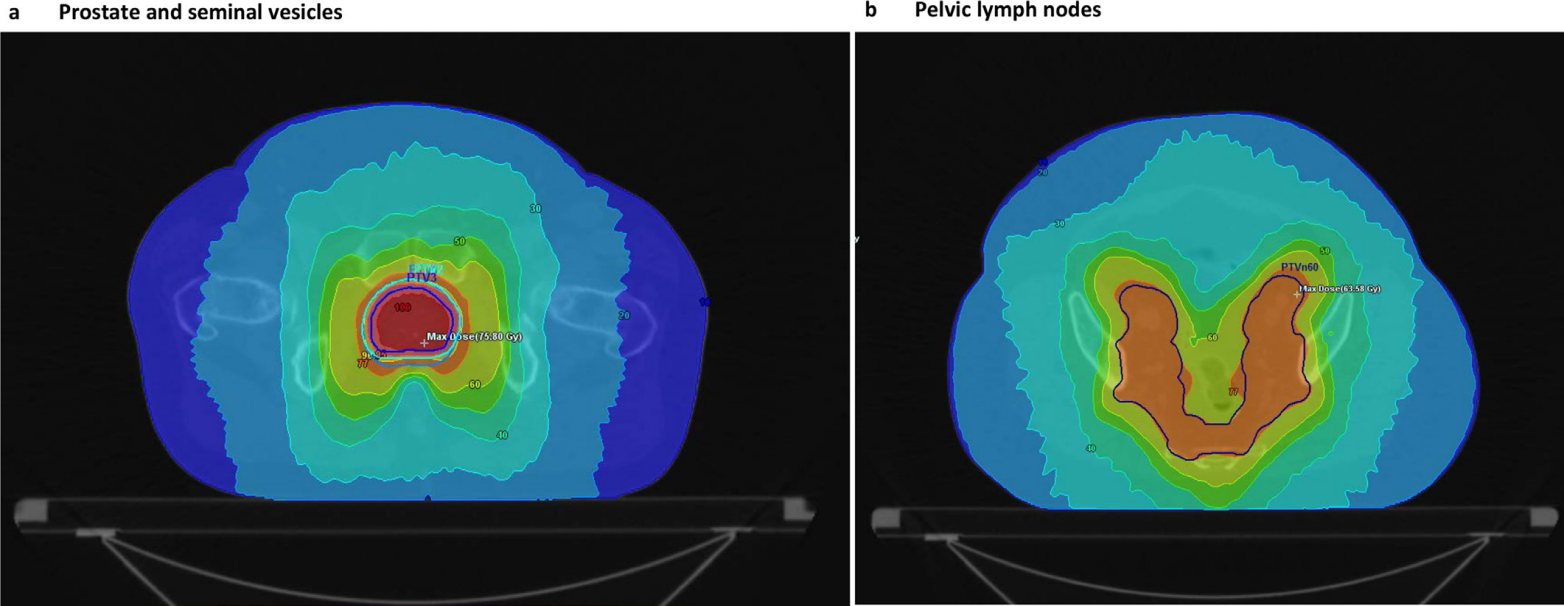
Example of a prostate and pelvic node radiotherapy treatment plan, showing the treatment volumes and radiation dose distribution.

All patients started on neo-adjuvant ADT for 3–6 months prior to PPLN EBRT and received 37 fractions of EBRT in one phase via SIB (Figure 2). The prostate and SV were treated to a maximum of 74 Gy EQD2, and the majority (96.7%) received a PLN EBRT dose of at least 43 Gy EQD2 (median dose 51 Gy EQD2). Only two patients (1.7%) received a PLN EBRT dose of <43 Gy EQD2 due to OAR constraints (Table 2). All patients had on-treatment reviews during EBRT to monitor and manage adverse events.

### Morbidity

The majority of patients (94%) had either no toxicities or mild adverse events recorded from PPLN EBRT (Table 3). The most common recorded toxicities were diarrhoea (41%, worst toxicity was Grade 2 = 10.7%) and cystitis (33%, worst toxicity was Grade 2 = 12.1%), with symptoms resolving after EBRT. Only two patients (1.7%) had toxicities of Grade 3 or more recorded; one patient had Grade 3 proctitis that persisted beyond EBRT, and another had Grade 3 haematuria that eventually settled following treatment. No major bowel complications requiring surgery were recorded.

**Table 3.** Toxicities of PPLN EBRT

Toxicities				
	**No toxicities**	**RTOG Grade 1–2**	**RTOG Grade 3–5**	**Unknown**
**ALL ADVERSE EVENTS**	39 (32%)	75 (62%)	2 (2%)	5 (4%)
**GENITOURINARY**				
Cystitis	77 (64%)	40 (33%)	0 (0%)	4 (3%)
Haematuria	112 (93%)	3 (2%)	1 (1%)	5 (4%)
Urethral stricture	113 (94%)	3 (2%)	0 (0%)	5 (4%)
**GASTROINTESTINAL**				
Diarrhoea	66 (55%)	50 (41%)	0 (0%)	5 (4%)
Proctitis	105 (87%)	10 (8%)	1 (1%)	5 (4%)
Recto-anal stricture	116 (96%)	0 (0%)	0 (0%)	5 (4%)
Bowel complications	102 (84%)	14 (12%)	0 (0%)	5 (4%)

EBRT, external beam radiotherapy; PPLN, prostate and pelvic nodal.

### Outcomes

Following EBRT, 118 patients had post-treatment PSA values recorded. 59 patients (48.8%) had undetectable nadir PSA levels within 1 year, whilst 49 patients (40.5%) had nadir PSA values between 0.1 and 2 ng ml^−1^ (median 0.4). 10 patients (8.3%) had nadir PSA values ≥2 ng ml^−1^ (median 3.84).

Eighty-eight patients (72.7%) were still alive with a median follow-up of 56 months (range 1–145 months). The 5-year OS was 74.4% (95% CI: 66.3–83.5%), whilst 5 year PCSS was 89.1% (95% CI: 82.6–96.0%). 33 patients (27.2%) had died at the time of analysis. 19 deaths were due to prostate cancer, whilst the remaining 14 deaths were from unrelated causes; namely other malignancies (*n* = 9), sepsis (*n* = 2), and unrelated organ failures (*n* = 3).

**Figure 3.**
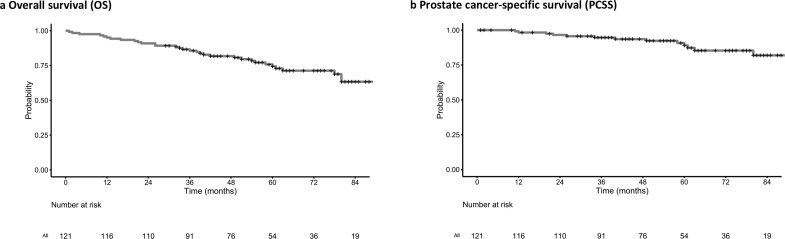
Kaplan–Meier survival estimates for (a) OS, and (b) PCSS in cN1 prostate adenocarcinoma patients receiving prostate and pelvic nodal EBRT. EBRT, external beam radiotherapy; OS, overall survival; PCSS, prostate cancer-specific survival.

**Figure 4.**
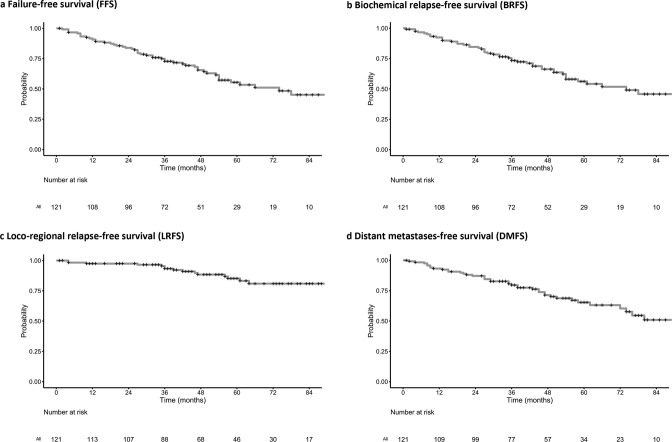
Kaplan–Meier survival estimates for (a) FFS, (b) BRFS, (c) LRFS, and (d) DMFS in cN1 patients receiving prostate and pelvic nodal EBRT. BRFS, biochemical relapse-free survival; DMFS, distant metastases-free survival; EBRT, external beam radiotherapy; FFS, failure-free survival; LRFS, loco-regional recurrence-free survival.

Forty-eight patients (40.5%) had experienced treatment failure, with the majority (*n* = 40) becoming castrate-resistant during adjuvant ADT. Median PSA on recurrence was 5.28 ng ml^−1^. The breakdown of treatment failure is presented in Table 4. Five-year FFS was 55.4% (95% CI: 45.9–66.9%); with 5-year BRFS at 56.2% (95% CI: 46.7–67.7%), 5-year LRFS at 85.2% (95% CI: 77.8–93.2%), and 5-year DMFS at 65.4% (95% CI: 56.1–76.1%) (Figures 3 and 4). Median FFS was 74 months. 28 patients developed biochemical relapse followed by distant metastases, whilst only 5 patients developed locoregional recurrence with or without biochemical relapse. Of the 39 patients with distant metastases, 12 developed bone metastases, 22 developed visceral metastases, and 5 developed both. The results of univariate analyses are summarised in Table 5, with PLN EBRT dose ≥55 Gy (EQD2) associated with increased LRFS (*p* = 0.021), but Gleason grade Group 4 to 5 and detectable nadir PSA ≥2 ng ml^−1^ associated with worse BRFS (*p* = 0.008) and DMFS (*p* = 0.019).

**Table 4.** Details of cN1 prostate adenocarcinoma patients who developed treatment failure after completing PPLN EBRT

Number of patients(%)	No relapse	Biochemical relapse only	Locoregional recurrence	Distant metastases
No biochemical relapse	72	5	0	1
Biochemical relapse			5	28
Biochemical relapse & locoregional recurrence				10

**Table 5.** Univariate analyses of clinicopathological parameters

Factor	n	5-year OS	*p*	5-year PCSS	*p*	5-year FFS	*p*	5-year BRFS	*p*	5-year LRFS	*p*	5-year DMFS	*p*
**T stage**													
T1 to T2	34	70.8%	–	89.1%	–	73.4%	–	76.5%	–	92.1%	–	79.7%	–
T3 to T4	87	72.0%	0.571	89.7%	0.523	50.4%	0.151	50.3%	0.114	83.3%	0.373	61.0%	0.157
**Initial PSA**													
<50 ng ml^−1^	77	68.92%	–	85.6%	–	60.8%	–	62.0%	–	88.0%	–	68.2%	–
>50 ng ml^−1^	43	83.6%	0.772	94.5%	0.443	48.8%	0.218	48.9%	0.104	83.2%	0.597	63.0%	0.772
Unknown	1	NA		NA		NA		NA		NA		NA	
**Nadir PSA post-EBRT**													
Undetectable	59	81.9%	–	100%	–	75.6%	–	77.4%	–	90.2%	–	90.3%	–
<2 ng ml^−1^	49	77.6%	0.555	84.9%	0.055	37.8%	0.001	37.6%	<0.001	84.3%	0.710	43.3%	0.001
≥2 ng ml^−1^	10	20.0%	<0.001	50.0%	<0.001	0%	<0.001	0%	<0.001	80.0%	0.045	0%	<0.001
Unknown	3	NA		NA		NA		NA		NA		NA	
**Gleason grouping**													
Gleason Group 1 to 3	34	76.8%	–	96.9%	–	74.7%	–	78.2%	–	82.2%	–	82.5%	–
Gleason Group 4 to 5	86	73.3%	0.391	86.2%	0.130	49.1%	0.017	48.9%	0.008	87.5%	0.494	58.3%	0.019
Unknown	1	NA		NA		NA		NA		NA		NA	
**Lymph node EBRT dose (EQD2)**													
≥55 Gy	59	69.8%	–	86.9%	–	63.9%	–	65.6%	–	95.7%	–	66.7%	–
43–54 Gy	58	80.7%	0.131	89.9%	0.818	50.6%	0.206	50.6%	0.138	74.8%	0.021	63.8%	0.515
<43 Gy	2	NA		NA		NA		NA		NA		NA	
Unknown	2	NA		NA		NA		NA		NA		NA	
**Duration of ADT**													
<2 years	28	74.3%	–	89.6%	–	45.4%	–-	45.5%	–	94.4%	–	61.8%	–
≥2 years	93	74.4%	0.980	88.9%	0.757	57.5%	0.586	58.5%	0.537	82.4%	0.688	66.9%	0.419
**Number of lymph nodes**													
≤3 lymph nodes	95	72.3%	–	87.6%	–	57.9%	–	58.9%	–	87.5%	–	67.0%	–
>3 lymph nodes	20	77.5%	0.351	93.3%	0.301	50.2%	0.700	50.2%	0.634	77.4%	0.202	56.1%	0.455

ADT, androgen deprivation therapy; EBRT, external beam radiotherapy; PSA, prostate-specific antigen.

### Subsequent management following treatment failure

Following treatment failure, 13 patients were treated with ADT only; 8 patients restarted ADT whilst 5 patients continued on their current ADT regimen. The median duration of ADT treatment alone on progression was 17.5 months (range 1–46 months). Two of these patients also received palliative EBRT. 31 patients received systemic treatment alongside ADT; 17 patients received 1 line of systemic treatment, 11 patients received 2 lines of systemic treatment, whilst 3 patients received 3 or more lines of systemic treatment. Four patients did not receive either ADT or systemic treatment and instead were treated with further surgery, palliative EBRT, or best supportive care.

## Discussion

The cohort of cN1 patients are at high risk of disease progression.^15^ The treatment paradigm ranges between locally advanced cN0 patients or metastatic disease. Despite the scarcity of randomised prospective evidence, many clinicians are advocating PPLN EBRT in addition to ADT,^16^ and this is now endorsed in multiple guidelines.^17–19^ We selected a cohort of cN1 patients treated with this approach and demonstrated comparable efficacy and toxicity to other contemporary published studies.

The benefits of EBRT in addition to ADT in cN1 patients has been demonstrated within the STAMPEDE control arm and is accepted within most UK centres. A retrospective subset analysis of 177 cN1 patients from the STAMPEDE control arm included 71 patients that received EBRT and ADT. EBRT volumes included the PLN treated to 45–50 Gy (EQD2) in 58 patients (82%). Estimated outcomes at 5 years were 65% FFS and 82% OS, demonstrating improvement over ADT alone.^4^ A much earlier trial, the RTOG 85–31 study, also published a retrospective subset analysis of 173 cN1 patients of which 98 patients (56.6%) received PPLN EBRT in addition to ADT. PLN were treated up to the L5-S1 interspace and received a dose of 44–46 Gy (EQD2). Outcomes at 5 years were 54% PSA control <1.5 ng ml^−1^ and 72% OS for combination treatment, which was improved compared to EBRT alone.^20^ Other published retrospective series also demonstrate improved survival outcomes, including large scale retrospective database analyses.^21–25^ However, these studies did not describe EBRT to PLN. Furthermore, there are no retrospective or prospective studies comparing prostate only EBRT against PPLN EBRT in cN1 patients on ADT.

A list of contemporary studies describing the use of PPLN EBRT in cN1 patients is listed in Table 1. The outcomes for 5-year FFS ranges from 50 to 80%, and is comparable to our findings. Of course, caution must be applied when comparing results due to the heterogeneity of these studies.^26–30^ For example, Lilleby et al included cN0 patients and cN1 patients with less than 3 PLN^26^ whilst Tsumura et al included oligometastatic M1 patients.^27^ Furthermore, EBRT dose fractionation was also different, with some delivering up to 55 Gy (EQD2) whilst others employed dose escalation either to the whole pelvis or via local boost to involved PLN. There were differences in time on ADT as well, although all described treatment for at least 12 months. Despite this, all demonstrated the benefits of adding PPLN EBRT alongside ADT in cN1 patients.

Our cN1 patient cohort are similar in clinicopathological parameters to the STAMPEDE cN1 cohort, except for more WHO performance status ≥1 patients involved in our study (Table 2). Overall, PPLN EBRT was well tolerated, with only two incidences of Grade 3 adverse events recorded following a median follow-up of 56 months. Our 5-year outcomes of FFS and OS are comparable to that of STAMPEDE, but we also described excellent 5-year PCSS and LRFS of 89.1 and 85.2%, respectively. There was prolongation of the failure-free period following PPLN EBRT, with a median FFS of 74 months. Majority of patients in our study (59.5%) did not experience treatment failure, and the efficacy of PPLN EBRT was seen with few locoregional recurrences, particularly with PLN EBRT doses of ≥55 Gy (EQD2). Those with more extensive disease (*e.g.* T3–T4 disease, >3 pelvic lymph nodes) did not demonstrate poorer outcomes, indicating that local treatment was adequate in these patients. Instead, most treatment failures involved biochemical relapse followed by distant metastases (Table 4), and most had either Gleason grade grouping 4 to 5, had progressed during adjuvant ADT, and/or had nadir PSA ≥2 ng ml^−1^ post-EBRT. This suggests a substantial subset of patients with either an aggressive tumour biology and/or presence of micro-metastatic disease at diagnosis not detected by staging imaging at the time. Most of these patients went on to have systemic treatment in addition to ADT afterwards.

From here, the paradigm of future research in cN1 patients continues to evolve. Dose escalation in PPLN EBRT treatment is now possible with the rapid adoption of IMRT, allowing significant sparing of OARs.^31,32^ As we demonstrated, there is improved LRFS with higher PLN EBRT doses ≥55 Gy (EQD2), but this did not impact BRFS, DMFS, OS, or PCSS. This is being further investigated in the PRIME trial using moderate and extreme hypofractionation EBRT to the prostate and PLN in high risk cN0 and cN1 patients alongside boost to gross nodal disease.^33^ As the few patients who died or had disease progression in our study mostly developed biochemical recurrence followed by distant metastases, this was the driver to poor outcomes. Several trials have sought to mitigate this with the addition of systemic treatment. The GETUG-12 trial and the follow-up of non-metastatic patients from STAMPEDE showed benefit in FFS after adding Docetaxel following EBRT in cN0 and cN1 patients,^34,35^ whilst a recent STAMPEDE publication demonstrated increased DMFS with the addition of Abiraterone in high risk non-metastatic prostate adenocarcinoma, of which 39% were cN1 patients.^36^ The majority of patients involved here, however, received PPLN EBRT in addition, and we believe this should be the case for most cN1 patients, due to good local control and significant prolongation of the failure-free period.

A strength of our study is that we describe one of the largest retrospective series of cN1 patients treated with PPLN EBRT in addition to ADT, with outcomes in keeping with those seen in the STAMPEDE and RTOG 85–31 cN1 cohorts.^4,20^ Furthermore, we described consistent EBRT dose fractionations with defined treatment volumes and dose constraints. We also demonstrated similar toxicity to PIVOTAL,^8^ with most common adverse events recorded as mild and most patients making a full long term recovery (Table 3). The limitations to our study include biases from retrospective analyses and lack of randomisation, as inherent in retrospective single centre series.^37^ We also lacked a cohort of cN1 patients undergoing ADT and/or prostate EBRT alone for comparison. Due to smaller patient numbers, multivariate analyses of prognostic factors were not possible. Toxicity data were also reliant on accurate documentation by clinicians during follow-up, and no formal proforma was used for documentation. Due to these limitations, larger multicentre prospective studies will normally be required to confirm our findings. However, based on the difficulties RTOG 96–08 faced in recruiting patients, it may not be possible to answer this question within a randomised controlled trial. In this context, our study provides real-world data in the management of cN1 patients receiving PPLN EBRT.

## Conclusion

In conclusion, we describe favourable outcomes following PPLN EBRT alongside ADT in one of the largest published cohort of prostate adenocarcinoma patients with cN1 disease. In the absence of randomised control trials investigating PPLN EBRT in such patients, we provide real-world data of this treatment modality, with prolonged LRFS and PCSS seen at 5 years. Treatment was well tolerated, time to disease progression was prolonged, and the majority of patients were rendered failure-free afterwards. Based on these results, prostate adenocarcinoma patients with cN1 disease should be considered for PPLN EBRT, in addition to ADT.
